# Antimnemonic effects of schemas in young and older adults

**DOI:** 10.1080/13825585.2015.1048774

**Published:** 2015-05-18

**Authors:** Stephen P. Badham, Elizabeth A. Maylor

**Affiliations:** ^a^ Department of Psychology, University of Warwick, Coventry, UK

**Keywords:** aging, schemas, associative memory, cued recall, picture recognition

## Abstract

Schema-consistent material that is aligned with an individual’s knowledge and experience is typically more memorable than abstract material. This effect is often more extreme in older adults and schema use can alleviate age deficits in memory. In three experiments, young and older adults completed memory tasks where the availability of schematic information was manipulated. Specifying nonobvious relations between to-be-remembered word pairs paradoxically hindered memory (Experiment 1). Highlighting relations within mixed lists of related and unrelated word pairs had no effect on memory for those pairs (Experiment 2). This occurred even though related word pairs were recalled better than unrelated word pairs, particularly for older adults. Revealing a schematic context in a memory task with abstract image segments also hindered memory performance, particularly for older adults (Experiment 3). The data show that processing schematic information can come with costs that offset mnemonic benefits associated with schema-consistent stimuli.

It has long been argued that the storage of information in memory is influenced by schematic knowledge, which defines rules and structure for the internal representation of information about the world (Bartlett, 1932). Schema theories postulate that a guiding schematic knowledge framework influences memory storage to form knowledge-consistent, expectation-confirming representations of an experience (Alba & Hasher, 1983). Many experiments have shown positive mnemonic properties of schema use in memory tasks. For example, Bransford and Johnson (1972) asked participants to listen to passages of text (vague instructions about doing laundry), where in one condition, the context of the passage (doing laundry) was revealed before listening, and in two other conditions, the context was revealed after listening or not at all. Participants aware of the schematic context before listening showed better understanding of the passage and better memory for the passage than did unaware participants. Other studies have shown that material relatable to schematic knowledge is more easily remembered than novel material. For example, words are easier to remember than nonwords and foreign words (Hulme, Maughan, & Brown, 1991), and images of faces are easier to remember than abstract images (Goldstein & Chance, 1971). Furthermore, studies have shown that information relevant to an individual’s expertise is more easily memorized (Arbuckle, Vanderleck, Harsany, & Lapidus, 1990; Chase & Simon, 1973; Miller, 2003).

In a recent review, Umanath and Marsh (2014) discussed age differences in the use of prior knowledge in memory tasks. Older adults show deficits in a large range of episodic memory tasks relative to young adults (Naveh-Benjamin & Ohta, 2012; Zacks, Hasher, & Li, 2000). Umanath and Marsh highlighted a variety of studies showing that older adults can be more influenced by schematic knowledge than young adults. This can alleviate age deficits in memory for material consistent with schematic knowledge (e.g., Badham, Estes, & Maylor, 2012; Castel, 2005; Shi, Tang, & Liu, 2012) and can have a greater detrimental effect on older adults’ memory relative to young adults’ memory for material inconsistent with schematic knowledge (e.g., Dalla Barba, Attali, & La Corte, 2010; Ruch, 1934). The current study focuses on age differences in memory for material with versus without schematic support.

Older adults do not always show greater benefits of schematic knowledge application in memory tasks compared to young adults (e.g., Arbuckle et al., 1990; Morrow, Leirer, Carver, & Tanke, 1998). However, with associative memory tasks, older adults seem to show reliably reduced age deficits for conditions in which schemas can be applied to the associations (e.g., Naveh-Benjamin, Hussain, Guez, & Bar-On, 2003). Much evidence suggests that older adults have a specific deficit for associative/context memory compared to item/source memory (Naveh-Benjamin, 2000; Old & Naveh-Benjamin, 2008; Spencer & Raz, 1995). For example, Naveh-Benjamin (2000) found that older adults can remember individual stimuli (e.g., words within word pairs) relatively well, but they show significantly larger age deficits for associations between items in memory (e.g., combinations of words within word pairs). A key factor alleviating this age-related associative deficit is the application of schematic knowledge to associations. In studies by Naveh-Benjamin, Hussain, et al. (2003) and Badham et al. (2012), age deficits in memory for associations between words within word pairs were reduced when those pairs contained semantically related words (e.g., flashlight–candle) compared to when they were unrelated (e.g., pillow–candle). Age deficits in associative memory are similarly reduced when word pairs consist of highly associated words based on free association norms (e.g., Kausler & Lair, 1966; Shaps & Nilsson, 1980) and for related pairs of pictures (Smith, Park, Earles, Shaw, & Whitinga, 1998).

The encouragement of strategic processing has also been shown to alleviate the age-related associative deficit, and it may be the case that schema use and strategic processing may alleviate the deficit by similar mechanisms. In incidental memory tasks, Glisky, Rubin, and Davidson (2001) showed that age deficits in source memory were eliminated when participants were oriented toward the association between items and contexts at encoding (participants were asked how well a chair matched the décor of a room at encoding in Experiment 3, or how likely a voice was to speak a sentence in Experiment 4). These data are in line with Naveh-Benjamin’s (2000) Experiment 2, where the age-related associative deficit was reduced under incidental encoding of associations between words within word pairs, compared to intentional encoding of those associations. When not intentionally encoding information (i.e., without deliberate employment of memory strategies), age deficits in associative memory were alleviated, consistent at least with the notion of a strategy production (and/or implementation) deficit in older adults for intentional associative memory tasks. This view is supported by data from Dunlosky and Hertzog (2001), who identified strategy production deficits in older adults in a paired associates task, with age deficits in memory reduced when strategies were explained to participants prior to encoding. This was investigated further by Naveh-Benjamin, Brav, and Levy (2007), who showed that encouraging older adults to use memory strategies (sentence generation) with word pairs at encoding or at encoding and retrieval alleviated or eliminated (respectively) the age-related associative deficit (i.e., age deficits in associative memory became comparable to age deficits in item memory).

The current study aimed to investigate how orienting the processing of memory stimuli toward processing based on schematic knowledge can influence memory and, more specifically, age deficits in memory. Experiments 1 and 2 investigated schema application in associative memory tasks in order to establish if highlighting schematic properties of stimuli could aid older adults in the same way as highlighting effective memory strategies. Experiment 3 investigated more naturalistic schema application in the recognition of images, where linking abstract memory stimuli to existing schemas was facilitated by picture recognition.

## Experiment 1

Young and older participants were presented with pairs of words to encode before completing a cued recall task, where they were shown the left word of each pair and were asked to recall the word presented alongside it. In a schema-present condition, categories were shown during encoding to indicate a nonobvious relation between the words of each pair. In a schema-absent condition, these categories were not given. Encoding speed was also manipulated, because previous research has shown that strategy implementation can be more successful with slower compared to faster encoding speeds in young and older adults (Dunlosky & Hertzog, 1998). It was hypothesized that providing access to a schema would support memory, particularly in older adults who can show greater susceptibility to schematic effects and greater benefits from strategy instructions.

### Method

#### Participants

Thirty-two young and 32 older adults took part in the experiment.^1^ Young participants were recruited from the University of Warwick and received either £6 or course credit. Older participants were all living independently and were recruited from an age study volunteer panel populated by local advertisements; they each received £10 toward their travel expenses. All participants were native English speakers except for three young adults.

Background information on participants is summarized in Table 1. Young and older participants did not differ significantly in their years of education, *t* < 1. To assess cognitive functioning, participants completed the Digit Symbol Substitution test from the Wechsler Adult Intelligence Scale – Revised (Wechsler, 1981), as a measure of processing speed, and the multiple-choice part of the Mill Hill vocabulary test (Raven, Raven, & Court, 1988), as a measure of crystallized intelligence. The results were consistent with the literature (e.g., Salthouse, 2010), with young adults showing higher speed but lower vocabulary than older adults, *t*(57.25) = 11.48 and *t*(62) = −5.58, respectively.

**Table 1.** Background details for participants in Experiments 1–3.

	Experiment 1†		Experiment 2		Experiment 3†	
Variable	Young	Older	Young	Older	Young	Older
*N* (M/F)^1^	32 (14/18)	32 (14/18)	31 (5/26)	30 (7/23)	32 (15/17)	32 (13/19)
Age range	18–28	64–84	18–20	66–88	18–26	64–84
Mean age (*SD*)	21.0 (2.2)	74.0 (6.1)	19.1 (0.5)	74.8 (6.4)	20.7 (1.8)	73.6 (5.8)
Mean years of education (*SD*)	14.8 (1.8)	14.9 (2.4)	14.0 (0.7)	14.8 (4.2)	14.7 (1.8)	15.0 (2.5)
Speed (*SD*)^2^	76.0 (8.6)	46.6 (11.6)*	69.8 (11.7)	50.0 (10.8)*	76.0 (8.6)	47.2 (11.2)*
Vocabulary (*SD*)^3^	18.8 (3.9)	24.2 (4.0)*	16.8 (2.5)	23.4 (3.5)*	18.9 (3.8)	24.3 (3.9)*

Notes: ^1^ Number of participants whose data were included in the analyses (males/females).

^2^ Mean information processing speed (and standard deviation) based on the Digit Symbol Substitution test (Wechsler, 1981).

^3^ Mean vocabulary score (and standard deviation) based on the multiple choice section of the Mill Hill vocabulary test (Raven et al., 1988); maximum score = 33.

*Older adults significantly different from young adults, *p* < .001.

†The majority of participants in Experiments 1 and 3 were the same individuals.

#### Materials

One hundred and thirty-six words were selected from the English lexicon project (Balota et al., 2007) to form 68 word pairs. The words were all two-syllable nouns of seven to nine letters in length, and they all had no orthographic, phonological, or phonographic neighbors. Words were selected to have their frequency of use in language within ±1 of the median (6.2) of log HAL (i.e., 5.2–7.2). Finally, words were selected to be non-plural, non-names, and non-offensive.

Words were manually entered into matched pairs that fit within 12 different categories with five or six pairs per category (see Appendix). Categories were a nonobvious grouping that linked the words of a pair together (e.g., for the pair *trespass–golfing*, the category was *actions*). The word pairs were placed into 17-pair lists with each list featuring six different categories (two or three pairs from each of six categories). Two versions of the experimental stimuli were formed, each with four lists of 17 pairs. The two versions used the same individual pairs but these were grouped into lists differently. Each of the four lists had a buffer pair at the beginning and end for which memory was not tested later. The two versions of the stimuli used the same pairs as buffers.

All memory stimuli were presented in black font on a white background and all materials were shown on a laptop computer running E-Prime 2.0 (Psychology Software Tools, Pittsburgh, PA, USA); the height of on-screen text corresponded to approximately 1° of viewing angle.

#### Procedure

Participants were shown pairs of words for a later cued recall test. Word pairs were presented in a lowercase font in random order and were studied for either 4 s per pair or 8 s per pair (fast vs. slow presentation). Each participant viewed schema-present and schema-absent lists for each of the presentation rates (i.e., a total of four study–test blocks). Participants received practice before both the schema-present and the schema-absent conditions in order to ensure that they were familiar with the task. The two short practice tests each replicated the full study–test procedure of a single experimental condition with independent stimuli and with just six trials.

In the schema-present condition, the category to relate the words of each pair was presented in uppercase immediately above each word pair. Participants were instructed to try to remember which words were presented together and were told that above each word pair was a clue that stated something that the words had in common. For each study pair, there was an interstimulus interval of 500 ms, where the screen remained blank. This was followed by the presentation of the category alone for 1000 ms, and then the category remained on the screen whilst the memory stimuli were presented for either 4 s or 8 s (fast or slow presentation). This allowed participants to process the category initially before memorizing each word pair. They were told that they could use this information if they wanted to in order to help memorize the associations. In the schema-absent condition, presentation was identical except the category labels were replaced by two lines of “XXXXXX”. Here, participants were instructed to ignore the Xs as they were not relevant to the memory test. Schema-present and schema-absent conditions were balanced across participants such that a given word pair was seen with a category by one participant and without a category by another participant at both encoding speeds. Categories used in the schema-present condition were not used in the schema-absent condition for each participant in order to prevent participants guessing the category labels in the schema-absent condition.

Between encoding and retrieval, there was a 30-s delay where participants were required to respond true or false (with keys “J” and “F”, respectively) to the correctness of simple numerical equations (e.g., 7 + 3 = 10 – true). These were presented in white font on a black background to differentiate them from the memory test.

For cued recall, participants were then shown the left word of each pair and were asked to verbally report the word that it was originally paired with whilst their voice was digitally recorded. (Note that no category labels were presented at test.) Participants were given as long as they needed to recall each word and the experimenter pressed a button once a response was made to present the next cue word. There was a 500-ms interstimulus interval in between each word. The cue words were selected in a random order.

For counterbalancing, participants viewed both schema-present lists or both schema-absent lists first. Within each of these two conditions, either the fast encoding or the slow encoding was presented first. There were also two versions of the stimuli so there were eight (2 × 2 × 2) counterbalancing conditions in total, with four young and four older participants assigned to each of these.

#### Data preparation

Participants’ responses to the cue words were coded as: (1) correct recall of the target word, (2) an incorrect intrusion (i.e., an incorrect word from either within or outwith the experiment), or (3) a “don’t know” response.

Response times (RTs) were also measured as the experimenter clicked a mouse button as soon as a word was recalled (the same experimenter tested every participant). RTs were not counted if the participant changed their answer after saying another word, if the participant pronounced the word very slowly whilst still recalling it, if the participant spoke before responding, if the participant responded after moving on to the next word, and finally if the experimenter had to ask the participant if they wanted to move on.

Throughout the article, standard null hypothesis tests are accompanied by estimating a Bayes Factor implemented through JASP computer software (Love et al., 2015). The Bayes Factor (*BF*_10_) provides an odds ratio for the alternative/null hypotheses (values < 1 favor the null hypothesis and values > 1 favor the alternative hypothesis). For example, a *BF*_10_ of 0.40 would indicate that the null hypothesis is 2.5 times more likely than the alternative hypothesis (see Jarosz & Wiley, 2014).

### Results and discussion

#### Accuracy

**Figure 1.**
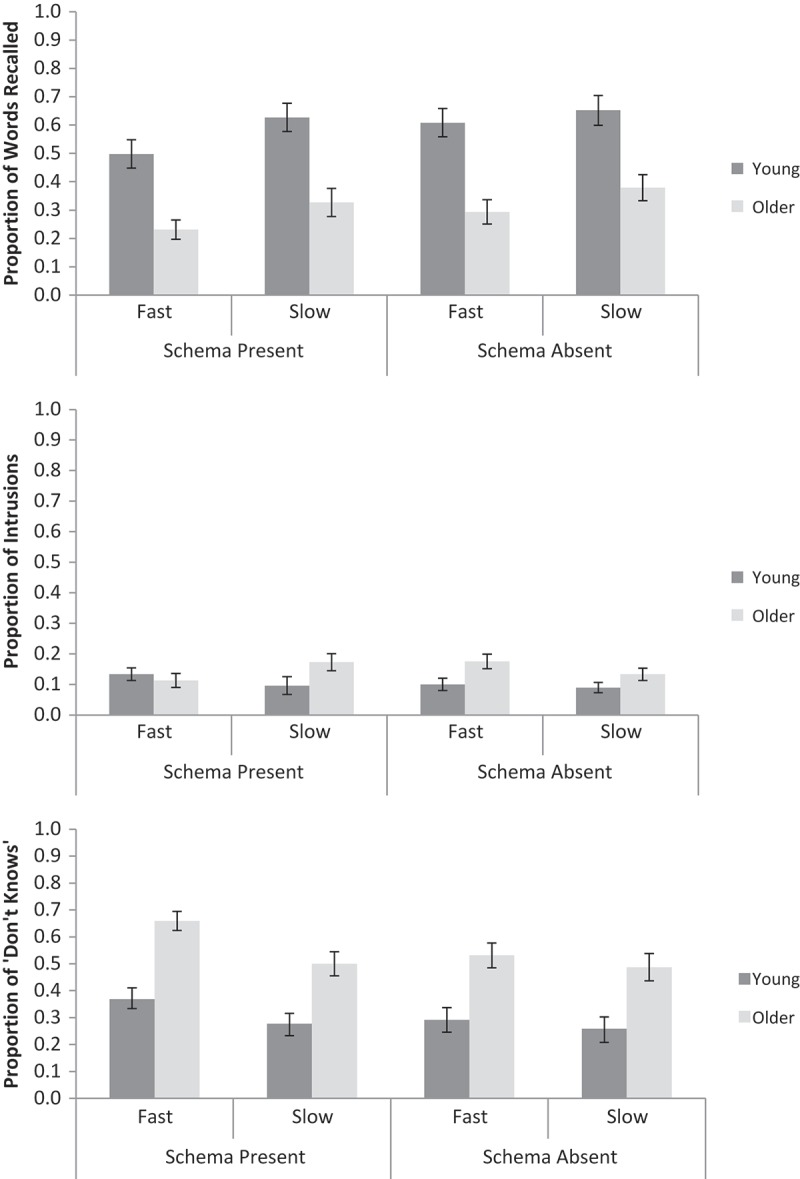
The proportion of words correctly recalled (top), intrusions (middle), and “don’t know” responses (bottom) for schema-present and schema-absent conditions, fast and slow encoding speeds, and young and older adults in Experiment 1. Error bars are ±1 *SE*.

A 2 (Age: young, older) × 2 (Schema presence: present, absent) × 2 (Encoding speed: fast, slow) repeated measures ANOVA was conducted on the proportion of correctly recalled words (see top panel of Figure 1 for means).^2^ Young adults recalled more words than older adults, *F*(1, 62) = 24.59, *MSE* = 0.22, *p* < .001, 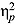  = .28, *BF*_10_ = 1247, consistent with general age deficits in memory. There was a main effect of schema presence, *F*(1, 62) = 9.98, *MSE* = 0.03, *p* < .01, 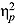  = .14, *BF*_10_ = 12.33, counterintuitively with words in schema-present lists being recalled *worse* than words in schema-absent lists. That is, when a schema was provided for participants, it hindered their memory performance. Slow encoding speed resulted in better recall than fast encoding speed, *F*(1, 62) = 22.22, *MSE* = 0.02, *p* < .001, 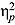  = .26, *BF*_10_ = 1511. There were no interactions (*F*s < 1.70; Age × Schema presence, *BF*_10_ **= 0.45; Age × Encoding speed, *BF*_10_ **= 0.40; Schema presence × Encoding speed, *BF*_10_ **= 0.79; Age × Schema presence × Encoding speed, *BF*_10_ **= 0.05).

The same ANOVA was conducted on the proportion of intrusions (see Figure 1, middle panel). Older adults produced marginally more intrusions than young adults, *F*(1, 62) = 3.17, *MSE* = 0.04, *p* = .08, 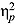  = .05, *BF*_10_ **= 0.38, but there were no main effects of schema presence (*F* < 1, *BF*_10_ **= 0.07) or encoding speed (*F* < 1, *BF*_10_ **= 0.08). There were no two-way interactions (*F*s < 3.02; Age × Schema presence, *BF*_10_ **= 0.07; Age × Encoding speed, *BF*_10_ **= 0.08; Schema presence × Encoding speed, *BF*_10_ **= 0.02), but there was a significant three-way interaction between age, schema presence and encoding speed, *F*(1, 62) = 7.96, *MSE* = 0.01, *p* < .01, 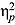  = .11, *BF*_10_ **= 0.08. Older adults produced more intrusions than young adults in all cases except for the fast schema-present condition.

For the proportion of “don’t know” responses (see Figure 1, bottom panel), older adults produced more than young adults, *F*(1, 62) = 22.14, *MSE* = 0.17, *p* < .001, 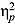  = .26, *BF*_10_ **= 677. There were more “don’t know” responses in the schema-present than in the schema-absent conditions, *F*(1, 62) = 7.45, *MSE* = 0.03, *p* < .01, 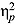  = .11, *BF*_10_ **= 17.07, which is consistent with the opposite pattern shown for correct responses. There were more “don’t know” responses for fast than for slow encoding speed, *F*(1, 62) = 21.16, *MSE* = 0.02, *p* < .001, 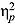  = .25, *BF*_10_ **= 922. There was also an interaction between schema presence and encoding speed, *F*(1, 62) = 6.81, *MSE* = 0.02, *p* < .05, 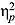  = .10, *BF*_10_ **= 4.29. For slow encoding speeds, “don’t know” responses were similar for schema-present and schema-absent but for fast encoding speeds there were more “don’t know” responses for schema-present than for schema-absent. This interaction suggests that the negative effects of schema presence can be overcome when encoding speed is slow, when participants have more time to process each association. There were no other interactions (remaining *F*s < 1.18; Age × Schema presence, *BF*_10_ **= 0.55; Age × Encoding speed, *BF*_10_ **= 0.68; Age × Schema presence × Encoding speed, *BF*_10_ **= 0.15).

#### Response times

**Figure 2.**
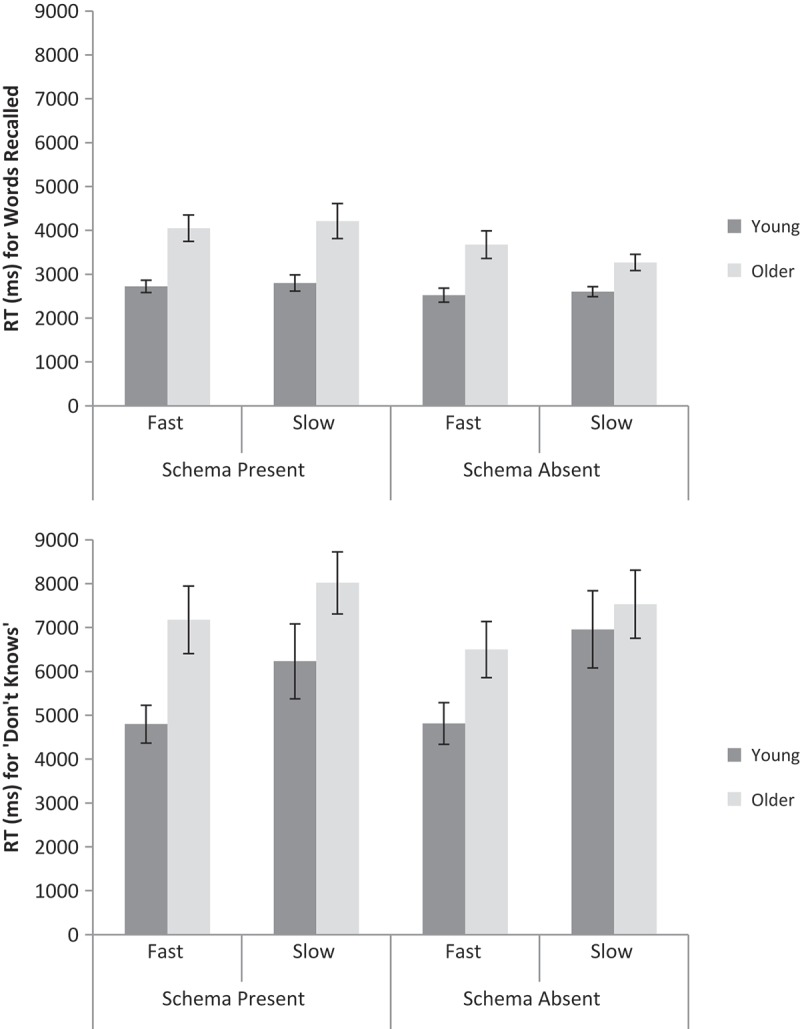
Mean RTs (based on medians for each participant) for correct (top panel) and “don’t know” (bottom panel) responses for schema-present and schema-absent conditions, fast and slow encoding speeds, and young and older adults in Experiment 1. Error bars are ±1 *SE*.

A 2 (Age: young, older) × 2 (Schema presence: present, absent) × 2 (Encoding speed: fast, slow) repeated measures ANOVA was conducted on the RTs for correctly recalled words based on the median RTs for each participant (see top panel of Figure 2 for means). Older adults (*n* = 23) responded slower than young adults (*n* = 28), *F*(1, 49) = 30.89, *MSE* = 2.12 × 10^6^, *p* < .001, 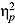  = .39, *BF*_10_ **= 5080. Correct responses in the schema-absent condition were faster than correct responses in the schema-present condition, *F*(1, 49) = 8.20, *MSE* = 1.14 × 10^6^, *p* < .01, 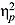  = .14, *BF*_10_ **= 3.77. This is in line with the accuracy data in that responses were faster in the more accurate condition. There was no main effect of encoding speed (*F* < 1, *BF*_10_ **= 0.08) and none of the interactions was significant (*F*s < 2.37; Age × Schema presence, *BF*_10_ **= 1.35; Age × Encoding speed, *BF*_10_ **= 0.09; Schema presence × Encoding speed, *BF*_10_ **= 0.79; Age × Schema presence × Encoding speed, *BF*_10_ **= 0.03). RTs for intrusions were not analyzed (only 11 young and 13 older adults had data in every cell). For “don’t know” responses (see bottom panel of Figure 2), older adults (*n* = 30) responded marginally slower than young adults (*n* = 24), *F*(1, 52) = 3.24, *MSE* = 4.26 × 10^7^, *p* = .08, 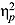  = .06, *BF*_10_ **= 0.79. There was no main effect of schema presence (*F* < 1, *BF*_10_ **=* *0.12). “Don’t know” responses occurred more quickly for fast than for slow encoding speeds, *F*(1, 52) = 20.75, *MSE* = 4.78 × 10^6^, *p* < .001, 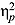  = .29, *BF*_10_ **= 1132. There were no interactions (*F*s < 2.32, Age × Schema presence, *BF*_10_ *= *0.19; Age × Encoding speed, *BF*_10_ **= 0.66; Schema presence × Encoding speed, *BF*_10_ **= 0.13; Age × Schema presence × Encoding speed, *BF*_10_ **= 0.03).

#### Summary

Providing young and older participants with a schematic link between to-be-associated words at encoding did not improve memory or alleviate age deficits in associative memory. Surprisingly, the schematic information significantly hindered accuracy and RTs suggesting that the processing of a schema directed participants’ resources away from effective memory processing.

Schemas can have a negative impact on memory performance. According to the false memory literature, activation of a schema can often lead to false memory for non-presented information that is consistent with the activated schema. Roediger and McDermott (1995) presented participants with a list of words to remember for a later free recall test, where all the words (e.g., thread, pin, eye, sewing, sharp…) were strong associates of a non-presented target word (e.g., needle). After studying such lists, a high proportion of participants falsely recalled the non-presented target word and often with high confidence. Schema-induced false memories have been found in a variety of memory studies (see Alba & Hasher, 1983; Umanath & Marsh, 2014; for reviews), including lists of facts about famous people (Sulin & Dooling, 1974), actions performed in scripts (Light & Anderson, 1983) and layouts of homes (Light & Anderson, 1983). These negative effects of schemas cannot be applied to the current result, as intrusions were few and were similar in the schema-present and schema-absent conditions; moreover, the use of multiple schemas within each block likely discouraged schema-based extrapolation.

Additionally, some studies find superior memory for schema-inconsistent information than for schema-consistent information. This occurs when schema-inconsistent information stands out because it does not fit with a context and has been demonstrated in a variety of distinctiveness paradigms (cf. Schmidt, 1991), including inconsistent character traits (Hess & Tate, 1991) and inconsistent objects within rooms (Mäntylä & Bäckman, 1992). This effect cannot be applied to the current data either, because the design did not include any schema-inconsistent stimuli (only schema-absent stimuli).

The negative effects of schema presence were likely due to the processing cost of applying the schematic information during encoding. Naveh-Benjamin, Craik, Guez, and Kreuger (2005) assessed the use of processing resources during encoding of related and unrelated word pairs in young and older adults (they manipulated processing resources by introducing a secondary task at encoding in Experiment 1). The encouragement of encoding strategies was also manipulated by suggesting sentence generation and mental imagery to half of the young and older participants. Encouraging strategies was more effective when participants’ attention was not divided, suggesting that processing resources are required for strategy implementation. That result is in line with the current data, where schema activation required processing resources. However, in the Naveh-Benjamin et al. (2005) study, the mnemonic effect of schema availability (better memory for related word pairs compared to unrelated word pairs) was not affected by available processing resources (see also Naveh-Benjamin, Guez, & Marom, 2003, Experiment 5, for similar results). This suggests that the current schemas were difficult to apply to the associations, possibly because the design required them to be nonobvious unless highlighted. Experiment 2 aimed to create more obvious schematic information by manipulating schema use with related and unrelated word pairs.

## Experiment 2

Given that schema use at encoding hindered paired associate performance in Experiment 1, Experiment 2 aimed to make the schema-orienting labels simpler and to make schematic information easier to use. Additionally, given that schematic information can have reconstructive properties at retrieval (Alba & Hasher, 1983) and that retrieval-based strategy manipulations have alleviated age deficits in memory (Naveh-Benjamin et al., 2007), Experiment 2 extended the investigation into the retrieval period. Young and older participants completed a paired associates task as in Experiment 1. Within each study list, half of the pairs were related and half were unrelated. In three conditions, different types of support were given to participants: In an encoding support condition, a label was presented during encoding to indicate if each pair was related or not. In a retrieval support condition, a label was presented during retrieval to indicate if each pair was related or not. In a control condition, no labels were provided.

### Method

#### Participants

Thirty-one young and 30 older adults took part in the experiment (see Table 1 for further details). They were all native English speakers and were recruited and rewarded in the same manner as in Experiment 1. Young and older participants did not differ significantly in their years of education, *t*(30.72) = 1.04. Young participants produced higher speed scores and lower vocabulary scores than older participants, *t*(59) = 6.86, and *t*(62) = −8.54, respectively.

#### Materials

Word pairs were constructed from stimuli used in Badham et al. (2012), where young and older adults also studied related and unrelated words for a cued recall test (the semantic, unrelated and target words in their appendix were used in this study). There were 45 “target” words which would always be used as the right word of a pair. For each target word, there was a semantically related word and an unrelated word (see Badham et al. for words used and their lexical statistics). This resulted in a total of 135 words capable of forming 45 related and 45 unrelated word pairs.

These words were used to randomly construct three study lists for each participant. The lists consisted of eight related and eight unrelated word pairs presented in a random order (i.e., a mixed list in terms of pair relatedness). Each target word was only used once, and correspondingly, no words were repeated across the three lists. The first and last pairs of words were used as buffers and were not cued in the retrieval phase. The buffers were one related and one unrelated pair (each placed randomly at either the beginning or the end of the list). Buffer words were not taken from the main set of words and were independently generated. Three practice lists were also produced using independent sets of six related and six unrelated word pairs.

All memory stimuli were presented in black font on a white background and all materials were shown on a laptop computer running E-Prime 2.0; the height of on-screen text corresponded to approximately 1° of viewing angle.

#### Procedure

Participants were shown pairs of words for a later cued recall test. Word pairs were presented in a lowercase font in random order for either 3 s per pair (young adults) or 6 s per pair (older adults). The different presentation durations were used to equate performance between young and older adults and were the same as used in previous studies (e.g., Badham et al., 2012; Naveh-Benjamin, 2000). In the encoding support condition, the relatedness of each pair was labeled by presenting the word *RELATED* or *UNRELATED* in uppercase on-screen above each word pair at study. For each study pair, there was an interstimulus interval of 500 ms, where the screen remained blank. This was followed by the presentation of the relatedness label for 1000 ms, and then the relatedness label remained on screen whilst the memory stimuli were presented. This allowed participants to process the relatedness initially before memorizing each word pair. Participants were instructed to try to remember which words were presented together and were told that above each word pair was a label that would inform them as to whether or not the words within each pair were related. In the retrieval support condition and in the control condition, presentation was identical except that the relatedness labels were replaced by “XXXXXXXX”. Here, participants were instructed to ignore the Xs as they were not relevant to the memory test. All stimuli were presented in black font on a white background.

Between encoding and retrieval, there was a delay period of 30 s requiring true/false responses to simple numerical equations as in Experiment 1.

For cued recall, participants were then shown the left word of each pair and were asked to verbally report the word with which it was originally paired whilst their voice was digitally recorded. In the retrieval support condition, the relatedness was indicated by presenting the word *RELATED* or *UNRELATED* above each retrieval cue during its presentation. This indicated if the cue was originally a member of a related or unrelated pair. In the encoding support and control conditions, the relatedness labels were replaced by “XXXXXXXX”. Participants were made specifically aware of this test format before encoding. Participants were given as long as they needed to recall each word and the experimenter pressed a button once a response was made to present the next cue word. There was a 500-ms interval in between each word. The cue words were selected in a random order.

Participants completed practice tests before each support condition to ensure that they were fully familiar with the procedure (the practice tests were presented in the same format as the condition to which they corresponded). Each participant received the three support conditions in different orders, resulting in six different possible test orders, which were counterbalanced across participants.

#### Data preparation

Correct responses, intrusions, and “don’t know” responses and RTs were computed for each participant as outlined in Experiment 1.

### Results and discussion

#### Accuracy

**Figure 3.**
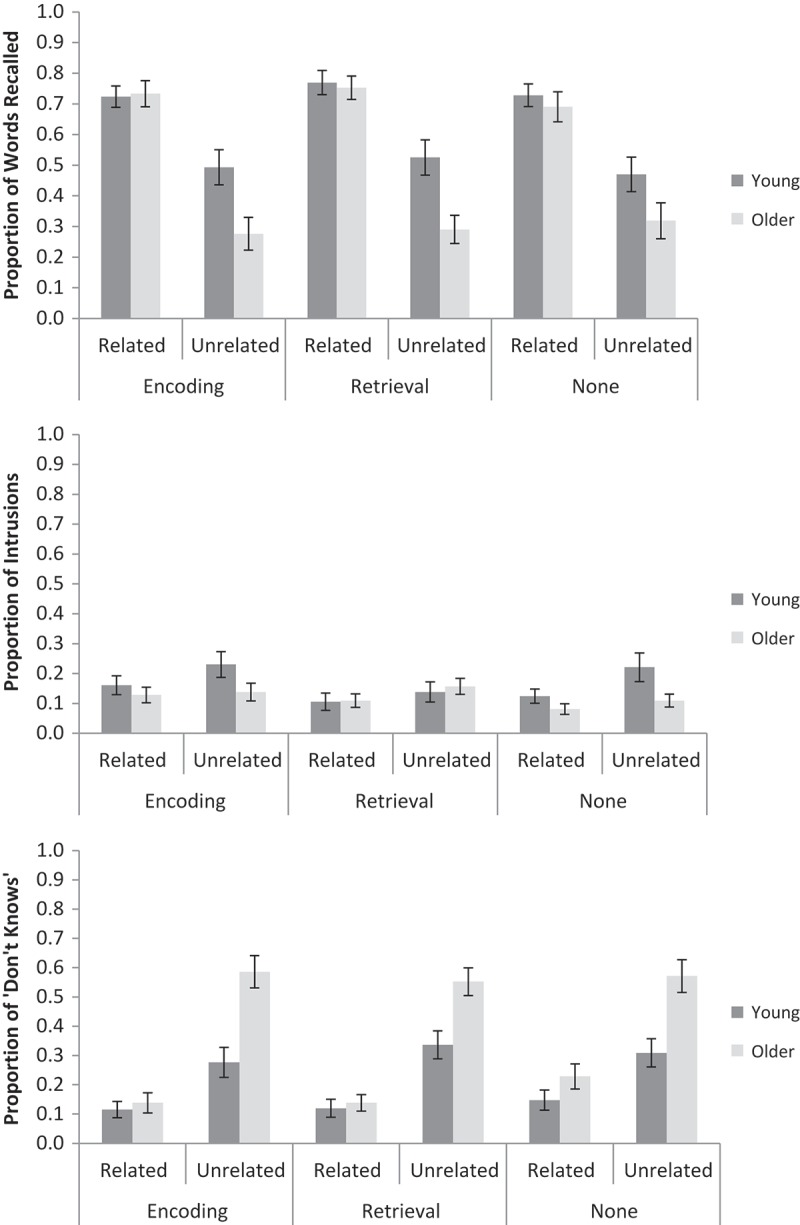
The proportion of words correctly recalled (top), intrusions (middle) and “don’t know” responses (bottom) for related and unrelated word pairs, for labels at encoding, retrieval or no labels, and for young and older adults in Experiment 2. Error bars are ±1 *SE*.

A 2 (Age: young, older) × 2 (Pair relatedness: related, unrelated) × 3 (Label condition: encoding, retrieval, none) repeated measures ANOVA was conducted on the proportion of correctly recalled words (see Figure 3 for means).^3^ Young adults recalled more words than older adults, *F*(1, 59) = 4.36, *MSE* = 0.25, *p* < .05, 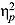  = .07, *BF*_10_ **= 2062. Surprisingly, there was no main effect of label condition, *F* < 1, *BF*_10_ **= 0.04, with similar recall performance no matter when or whether relatedness was indicated during the memory task. Related word pairs were recalled better than unrelated word pairs, *F*(1, 59) = 227.67, *MSE* = 0.05, *p* < .001, 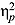  = .79, *BF*_10_ **> 10^10^. There was an interaction between age and pair relatedness, *F*(1, 59) = 17.30, *MSE* = 0.05, *p* < .001, 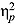  = .23, *BF*_10_ **= 4757, with age deficits in memory reduced for related pairs compared to unrelated pairs, replicating prior research with related and unrelated word associations (e.g., Badham et al., 2012; Naveh-Benjamin, Hussain, et al., 2003). There were no other interactions (*F*s < 1.23; Age × Label condition, *BF*_10_ **= 0.01; Pair relatedness × Label condition, *BF*_10_ *=* 0.01; Age × Pair relatedness × Label condition, *BF*_10_ **< 0.01).

The same ANOVA was conducted on the proportion of intrusions. There was no main effect of age, *F*(1, 59) = 1.93, *MSE* = 0.09, *ns*, 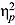  = .03, *BF*_10_ **= 0.43. Intrusions were less likely for related word pairs than for unrelated word pairs, *F*(1, 59) = 9.38, *MSE* = 0.03, *p* < .01, 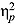  = .14, *BF*_10_ **= 14.51. The main effect of label condition was marginal, *F*(2, 118) = 3.00, *MSE* = 0.02, *p* = .05, 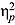  = .05, *BF*_10_ **= 0.28, and there was an age by label condition interaction, *F*(2, 118) = 4.35, *MSE* = 0.02, *p* < .05, 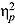  = .07, *BF*_10_ **= 0.55. Young and older adults made similar numbers of intrusions when there were labels at retrieval, but young adults made more intrusions in the other conditions. There were no other interactions (*F*s < 1.47; Age × Pair relatedness, *BF*_10_ **= 0.38; Pair relatedness × Label condition, *BF*_10_ **= 0.07; Age × Pair relatedness × Label condition, *BF*_10_ **= 0.01).

For “don’t know” responses, the results mirrored the correct-recall data. Older adults made more “don’t know” responses than young adults, *F*(1, 59) = 11.04, *MSE* = 0.19, *p* < .01, 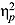  = .16, *BF*_10_ **= 2.70 × 10^7^. There was no main effect of label condition, *F*(2, 118) = 1.20, *MSE* = 0.04, *ns*, 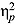  = .02, *BF*_10_ **= 0.05. Fewer “don’t know” responses were made for related word pairs compared to unrelated word pairs, *F*(1, 59) = 189.66, *MSE* = 0.04, *p* < .001, 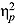  = .76, *BF*_10_ **> 10^10^, and there was an interaction between age and pair relatedness, *F*(1, 59) = 27.63, *MSE* = 0.04, *p* < .001, 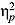  = .32, *BF*_10_ **= 6.28 × 10^6^. There were no other interactions (*F*s < 1.83; Age × Label condition, *BF*_10_ **= 0.03; Pair relatedness × Label condition, *BF*_10_ **= 0.03; Age × Pair relatedness × Label condition, *BF*_10_ **= 0.01).

#### Response times

**Figure 4.**
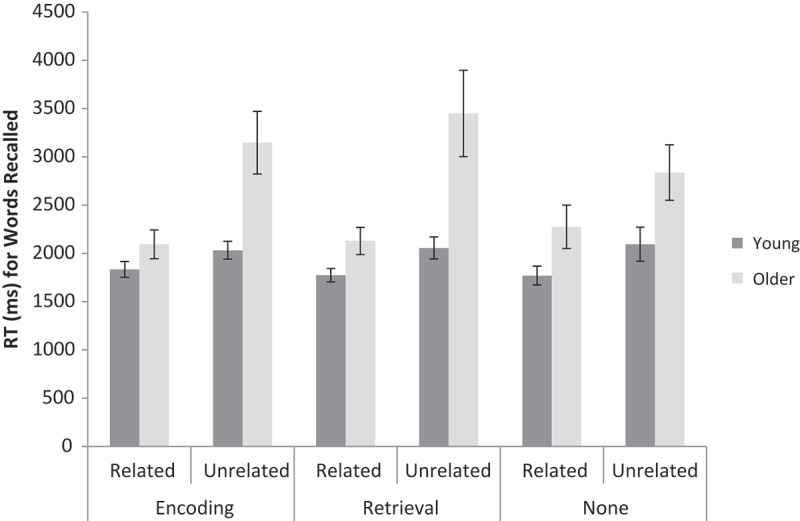
Mean RTs (based on medians for each participant) for correct responses for related and unrelated word pairs, for labels at encoding, retrieval, or no labels, and for young and older adults in Experiment 2. Error bars are ±1 *SE*.

A 2 (Age: young, older) × 2 (Pair relatedness: related, unrelated) × 3 (Label condition: encoding, retrieval, none) repeated measures ANOVA was conducted on RTs for correct recalls based on the medians for each participant (see Figure 4 for means). Older adults (*n* = 16) responded slower than young adults (*n* = 24), *F*(1, 38) = 15.14, *MSE* = 2.01 × 10^6^, *p* < .001, 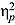  = .29, *BF*_10_ **= 8478. As with the accuracy data, there was no main effect of label condition, *F* < 1, *BF*_10_ **= 0.03. Responses to related word pairs were faster than responses to unrelated word pairs, *F*(1, 38) = 36.59, *MSE* = 6.12 × 10^5^, *p* < .001, 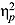  = .49, *BF*_10_ **= 9.10 × 10^8^, and there was an interaction between age and pair relatedness, *F*(1, 38) = 11.85, *MSE* = 6.12 × 10^5^, *p* < .01, 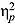  = .24, *BF*_10_ **= 741. Age differences in RTs were smaller for related word pairs than for unrelated word pairs, mirroring the accuracy data with faster responses in the more accurate condition. There was also a marginal triple interaction, *F*(2, 76) = 2.63, *MSE* = 6.44 × 10^5^, *p* = .08, 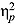  = .07, *BF*_10_ **= 0.02, suggesting that the age by pair relatedness interaction was somewhat stronger with than without labels. There were no other interactions (*F*s < 1.92; Age × Label condition, *BF*_10_ **= 0.03; Pair relatedness × Label condition, *BF*_10_ **= 0.03). The same analyses were not conducted on RTs for intrusions and “don’t know” responses, as there were not enough participants in each cell (eight or fewer) for meaningful analysis.

#### Summary

Manipulation of schema orientation had no effect on memory or age differences in memory. Performance was similar when relations between to-be-remembered words were highlighted at encoding, retrieval, or not highlighted at all. Unlike Experiment 1, highlighting relations between words did not hinder memory, indicating that schema orientation did not use processing resources. There were, however, age differences in schema use such that age deficits in memory were significantly alleviated when word pairs were related compared to unrelated, replicating prior research (Badham et al., 2012; Naveh-Benjamin, Hussain, et al., 2003) and also extending the result to a paradigm using mixed lists of related and unrelated pairs.

In a similar paired associates task, Froger, Bouazzaoui, Isingrini, and Taconnat (2012) found that the interaction between age and pair relatedness was absent when encoding strategies were encouraged but present when no encoding strategy was encouraged. The current data show almost the opposite pattern with significant age by relatedness interactions for accuracy when labels were present at encoding (*p* = .001, *BF*_10_ **= 52.64) and retrieval (*p* < .001, *BF*_10_ **= 114) but only a marginal interaction with the no labels condition (*p* = .10, *BF*_10_ **= 1.34). Furthermore, Naveh-Benjamin et al. (2005) found that encouraging encoding strategies did not influence the age by pair relatedness interaction.

Highlighting relations at encoding had no effect on memory, even though the relations themselves did improve memory. This indicates that the processing of sematic relations occurs automatically and is in line with data from Naveh-Benjamin’s laboratory (Naveh-Benjamin, Guez, et al., 2003; Naveh-Benjamin et al., 2005), where relatedness had similar effects on memory under full and divided attention. Perhaps more surprising is that indicating relations at retrieval had no beneficial effect on memory (except for possibly discouraging young adults from guessing – see middle panel of Figure 3); in this condition the label informed participants whether to search for a related or unrelated target for each cue, which presumably would have helped narrow down retrieval search processes. The absence of this effect suggests that any influence that retrieval labels may have on search efficiency is small.^4^ To avoid null effects of schema orientation, Experiment 3 aimed to ensure that schema activation could not occur in the schema-absent condition, and that in the schema-present condition, its activation would be natural and effortless.

## Experiment 3

**Figure 5.**
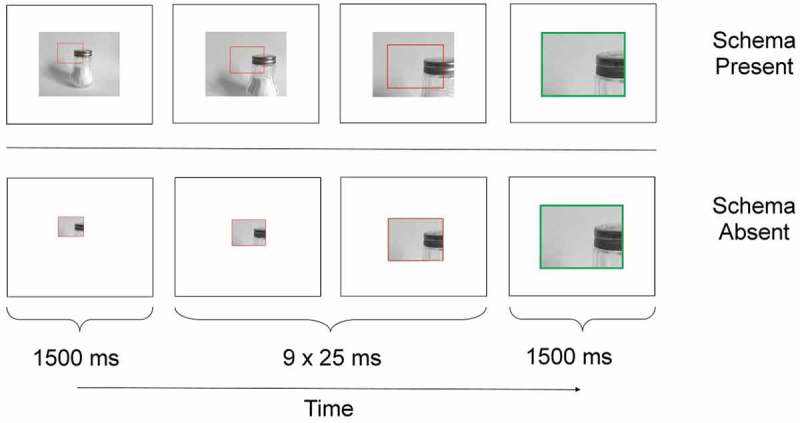
Schematic illustrations of example presentation trial screens for schema-present (top) and schema-absent (bottom) stimuli in Experiment 3. For each row, leftmost image indicates start screen, middle images depict two of nine zooming screens and rightmost image indicates end screen.

In order to further facilitate the ease of application of schematic knowledge, pictures were used as study material in Experiment 3. Small segments of images of common items were used such that the segments were abstract and unrecognizable when viewed out of context (e.g., a picture of the edge of a metal top taken from an image of a salt shaker; see Figure 5). In a schema-present condition, participants viewed the image segments in the context of the whole image (i.e., the full image was shown with the segment highlighted), and in a schema-absent condition, the image segment was viewed in isolation. This experiment was designed to encourage a natural and automatic association of the memory stimulus to a schematic context in the schema-present condition.

### Method

#### Participants

Thirty-two young and 32 older adults took part in the experiment.^5^ As indicated in Table 1, the majority also participated in Experiment 1, which took place earlier in the single testing session. Young and older participants did not differ significantly in their years of education, *t* < 1. Young participants produced higher speed scores and lower vocabulary scores than older participants, *t*(58.29) = 11.55, and *t*(62) = −5.54, respectively.

#### Materials

Eighty images of common man-made objects were collected from the internet and converted to grayscale. For each image, a segment was manually chosen such that if it were presented alone, it would not lead to identification of the object depicted in the original image. The image segments were the to-be-remembered stimuli during encoding. Each segment was yoked to a non-studied lure segment (similar in appearance) for use in a later old/new recognition test.

Figure 5 shows example encoding trials for schema-present and schema-absent conditions. For each encoding trial, the image segments were initially presented in their original size in relation to the full image from which they were taken. Following this, the viewpoint zoomed in until the segment occupied the entire viewing frame. Image segments were highlighted by a red box, but when the zooming stopped, the box changed to green. Each trial began with a 500-ms blank white screen. Then a segment was shown in its zoomed-out context for 1500 ms, it zoomed in for nine frames (each 25 ms long), and then remained zoomed in for 1500 ms. In the schema-present condition, the image segment was presented with the full image from which it was taken as a background; in the schema-absent condition, the image segment was presented identically but with no surrounding background. At all stages, the sizes and zooming trajectories were identical for a given segment in a schema-present and a schema-absent condition.

Memory was tested via an old/new recognition test: participants viewed the segments that they had seen before randomly mixed with lures matched to be similar to those segments. At test, the segments were displayed identically to the zoomed-in stage from encoding (i.e., end screen) but with a blue border instead of green to help participants differentiate between encoding and retrieval tasks.

Images were presented on a laptop computer running E-Prime 2.0 in the center of a 1024 × 768 pixel display within a frame of 512 × 384 pixels (zoomed-in study image segments and test stimuli filled this whole frame). This frame size corresponded to a height of approximately 10° of viewing angle for participants.

##### Stimulus selection

A validation task was conducted to ensure that participants could not easily guess the objects from the segments. Additionally, it was important to ensure that whole images were easily recognized so that in schema-present trials, the schema could easily be identified. The choice of lures was also assessed.

Fourteen independent participants (12 female; mean age = 18.3 years, *SD* = 0.47; mean education = 13.8 years, *SD* = 0.58) were shown whole images and zoomed-in segments of images (1500 ms each, image height 10° of viewing angle). They were asked to name each image if they were sure they knew what it was and to say “don’t know” if they were not sure what it was. The experimenter pressed a button for each of the possible results and an object was classed as named even if it was named incorrectly. This is because any named image would evidently have evoked a schema in the participant. Two versions of the stimulus preparation experiment were produced and both contained all of the lure image segments. However, a given image segment and the whole image from which it was taken were not shown to the same participant. This meant that half of the encoding stimuli were shown to each participant. Overall, each participant viewed 80 lure segments, 40 whole images, and 40 image segments.

Target image segments were excluded if more than one of the seven participants provided a name for the image, and whole images were excluded if more than one of the seven participants were unable to name the image. Lure images were excluded if more than four of the 14 participants provided a name for the image. This resulted in seven segment exclusions, three whole image exclusions, and 13 lure exclusions. The exclusion process also covered any images matched to the excluded images (i.e., if any one whole image, image segment, or matched lure from a group was excluded, then all three were excluded). In total, 20 sets of stimuli were removed, leaving 60 for use in the main experiment.

Target–lure similarity was also assessed using visual similarity rating scales. All 14 participants rated the matched target–lure image pairs as more similar in appearance than randomly paired targets and lures.

#### Procedure

The 60 sets of images were used to produce a block of 30 schema-present study stimuli and a block of 30 schema-absent study stimuli (images were randomly assigned to each block for each participant). The order of the blocks was counterbalanced between participants. Three of the excluded stimuli were used for a practice block that was displayed as schema-present during encoding. Participants first completed the practice session: During encoding, they were asked to remember the segments of images that were highlighted by the red box as they zoomed in on the screen. Then there was a short delay between encoding and retrieval, where participants were required to respond true or false to the correctness of simple numerical equations (as in Experiments 1 and 2). During retrieval, they were asked to use their two index fingers to press “J” for stimuli that they remembered from the encoding phase and to press “F” for stimuli that they had not seen before. Stimuli remained on screen until a response was made. After the practice, participants completed the two main conditions in the same way. The delay between encoding and retrieval, occupied by the numerical task, was fixed at 30 s. All 30 studied image segments and their matched lures were probed during retrieval, resulting in 60 retrieval trials for each block. At encoding and retrieval, all images were presented in random order for each participant.

### Results and discussion

#### Accuracy

**Figure 6.**
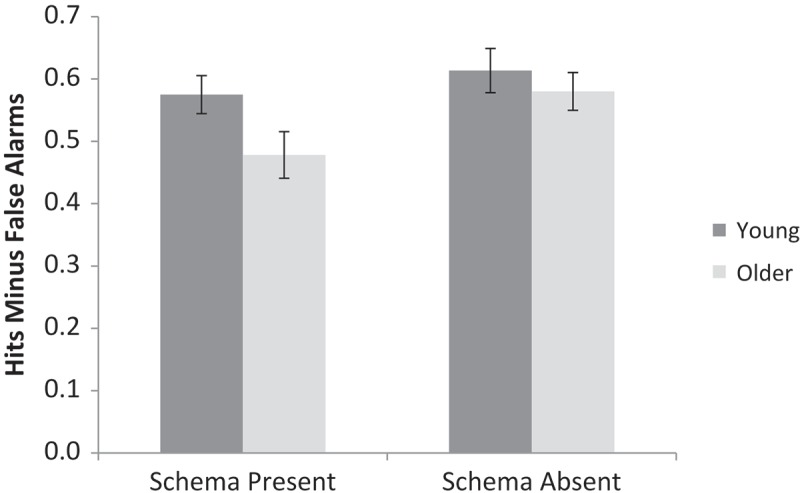
Proportion of hits minus proportion of false alarms for schema-present and schema-absent conditions for young and older adults in Experiment 3. Error bars are ± 1*SE*.

Accuracy was calculated as the proportion of hits minus the proportion of false alarms (e.g., as used by Naveh-Benjamin, 2000). A 2 (Age: young, older) × 2 (Schema presence: present, absent) repeated measures ANOVA was conducted on the accuracy data (see Figure 6 for means).^6^ There was no main effect of age, *F*(1, 62) = 2.53, *MSE* = 0.05, *p* = .12, 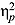  = .04, *BF*_10_ = 0.33, demonstrating relative age sparing of memory for pictorial stimuli, in line with prior research (e.g., Park, Puglisi, & Smith, 1986). There was a main effect of schema presence, *F*(1, 62) = 8.74, *MSE* = 0.02, *p* < .01, 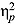  = .12, *BF*_10_ = 6.11, and no interaction, *F*(1, 62) = 1.78, *MSE* = 0.02, *p* = .19, 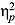  = .03, *BF*_10_ = 0.60. As in Experiment 1, participants counterintuitively performed better in the schema-absent condition than in the schema-present condition.

#### Response times

**Figure 7.**
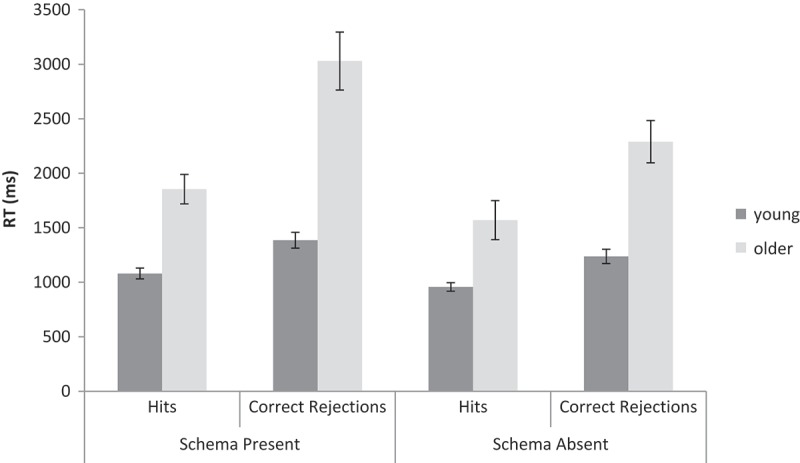
RTs for hits and for correct rejections for schema-present and schema-absent trials for young and older adults in Experiment 3. Error bars are ± 1*SE*.

RTs for correct responses only were analyzed in order to include as much data as possible. Median RTs were calculated for each participant for hits and for correct rejections. A 2 (Age: young, older) × 2 (Schema presence: present, absent) × 2 (Response type: hits, correct rejections) repeated measures ANOVA was conducted on the RTs (see Figure 7). Older adults responded slower than young adults, *F*(1, 62) = 30.06, *MSE* = 2.22 × 10^6^, *p* < .001, 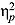  = .33, *BF*_10_ > 10^10^. In line with the accuracy data, responses to schema-present trials were slower than responses to schema-absent trials, *F*(1, 62) = 42.43, *MSE* = 1.58 × 10^5^, *p* < .001, 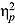  = .41, *BF*_10_ = 3.04 × 10^7^. There was an interaction between age and schema presence, *F*(1, 62) = 14.35, *MSE* = 1.58 × 10^5^, *p* < .001, 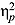  = .19, *BF*_10_ = 166, with a larger increase in RTs from schema-absent to schema-present for older adults compared to young adults. Thus, the presence of schematic information was more detrimental to older adults’ performance than to young adults’ performance. [Although the equivalent interaction for accuracy did not reach significance, paired *t*-tests on the accuracy data converged on a similar conclusion: schema-present was less accurate than schema-absent for older adults, *t*(31) = 3.07, *p* < .01, *BF*_10_ = 8.84, but not for young adults, *t*(31) = 1.13, *p* = .27, *BF*_10_ = 0.34.] Hits were faster than correct rejections, *F*(1, 62) = 110.02, *MSE* = 2.24 × 10^5^, *p* < .001, 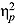  = .64, *BF*_10_ > 10^10^. There was an interaction between age and response type, *F*(1, 62) = 30.64, *MSE* = 2.24 × 10^5^, *p* < .001, 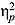  = .33, *BF*_10_ = 5.68 × 10^6^, with a larger increase in RTs from hits to correct rejections for older adults compared to young adults. There was also an interaction between schema presence and response type, *F*(1, 62) = 5.98, *MSE* = 1.55 × 10^5^, *p* < .05, 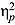  = .09, *BF*_10_ = 9.37, with a larger increase in RTs from hits to correct rejections for schema-present compared to schema-absent trials. The triple interaction was also significant, *F*(1, 62) = 4.76, *MSE* = 1.55 × 10^5^, *p* < .05, 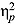  = .07, *BF*_10_ = 15.10; the overall pattern was that factors that reduced RTs did so cumulatively, leading to the interactions observed.

#### Summary

The pattern of results was similar to Experiment 1 and extends those findings to pictorial stimuli: Activation of schemas at encoding paradoxically hindered memory performance and slowed responses at retrieval. Experiment 3 also found that older adults suffered significantly more from schema activation than did young adults in terms of recognition RTs. (Note that a qualitatively similar age trend occurred for RTs in Experiment 1 – see Figure 2.)

The fact that providing schemas via pictures disrupted memory processes was surprising. Priming tasks show that pictures can be semantically processed and named rapidly (e.g., Sperber, McCauley, Ragain, & Weil, 1979), suggesting that they do not require large amounts of processing resources to be comprehended. Nonetheless, presentation of an entire image disrupted processing of a segment of that image, hindering memory for the segment *more* than any memory benefit attributable to schema activation. Integrating the full image with the segment was costly to memory, possibly because the integration required processing resources or because memory of the full image interfered with memory for the segment. The greater impact on older adults may be explained by the fact that they can take longer to recognize/name pictures (Burke & Shafto, 2008); alternatively, the full image may have caused greater interference to older adults’ segment memory (e.g., Hasher & Zacks, 1988). Overall, the main conclusion converges with that of Experiment 1, namely that schema processing can come with costs – even when no schema-inconsistent information is presented.

## General discussion

Unlike existing studies in the literature (Alba & Hasher, 1983; Umanath & Marsh, 2014), encouraging processing in terms of existing knowledge and experience did not aid memory in young and older adults. Across three experiments, orienting participants toward schema use hindered (Experiments 1 and 3) or made little difference (Experiment 2) to memory performance.

In Experiment 2, schematic information helped memory performance, but this effect occurred independently of any encouragement to use that information. Memory for word pairs was better for related than for unrelated words, and the age-related deficit in memory was significantly reduced when words were related. Labels orienting participants toward relations between words made no difference to their ability to form or retrieve associative memories, indicating that relations are processed automatically.

Experiments 1 and 3 showed poorer memory when schematic information was available to participants. These two experiments used different designs (Experiment 1: word association; Experiment 3: picture memory), thus demonstrating the effect across two very different contexts. In Experiment 1, participants studied word pairs where there was a nonobvious relation between the words. Memory for associations between those words was hindered in a condition where that relation was highlighted. Processing the relation *reduced* memory performance. This result seems to go against schema theory, where schema activation helps memory for material consistent with that schema (Alba & Hasher, 1983), and it is also inconsistent with levels of processing theory, where information that is processed more deeply is better remembered (Craik & Lockhart, 1972). It could be argued that because the relations were nonobvious, the relations did not fit well with commonly activated schemas. However, previous research has shown that schema activation can reduce age deficits in associative memory for pairs of words with no prior associations. For example, Badham et al. (2012) showed that integrative word pairs (e.g., monkey–foot, horse–doctor) alleviated age-related associative deficits to a similar extent as semantically related word pairs (e.g., paw–foot, sick–doctor), even though the integrative word pairs were extremely rare in language. Additionally, schema use also hindered memory in Experiment 3, which used more obvious schemas than Experiment 1. In Experiment 3, participants studied abstract segments of pictures either in the context of the original picture (schema-present) or alone (schema-absent). The original pictures were all of everyday items and so would fit well with commonly activated schemas. Schema presence hindered memory and particularly hindered older adults relative to young adults for recognition RTs.

In the introduction, we discussed how strategy production deficits may partly explain age differences in memory as studies encouraging strategy use alleviate age-related deficits in memory (e.g., Dunlosky & Hertzog, 2001; Naveh-Benjamin et al., 2007). The current data suggest that encouraging schema use does not always aid older adults’ memory in the same way as encouraging strategy use. It may be the case that whilst schema use can help older adults more than young, it may only do so when schema processing is automatic. This suggestion is in line with studies showing that relations between words have equivalent memory benefits under full and divided attention (Naveh-Benjamin, Guez, et al., 2003; Naveh-Benjamin et al., 2005).

The notion that prior knowledge may only be useful if it is easily implemented in memory studies is also shown in data from dementia research. Bäckman and Herlitz (1990) showed that both Alzheimer’s disease (AD) patients and controls had more knowledge of dated compared to contemporary famous faces but only the healthy older adults showed better recognition memory for the dated faces compared to contemporary faces. AD patients were, however, able to benefit from prior knowledge when recognizing dated faces compared to contemporary faces in a study by Lipinska, Bäckman, and Herlitz (1992), who asked participants to generate statements about the faces and provided names at encoding, allowing 1 minute of study time for each face. (In contrast, in Bäckman and Herlitz’s study, faces were passively viewed at a rate of 7 s per face.) Lipinska et al. argued that AD patients are less likely to spontaneously benefit from prior knowledge unless it is made easy to use. Following up this research, Lipinska and Bäckman (1997) also found that orienting AD patients towards semantic categories during encoding led to their utilization of that information during retrieval. But often dementia studies show that patients fail to utilize methods of cognitive support in episodic memory tasks (see Bäckman, Mäntylä, & Herlitz, 1990, for a review). This further indicates that prior knowledge manipulations must consider ease of application of that knowledge, particularly in AD research – and perhaps especially so, given the impaired semantic (prior knowledge) access in AD patients (e.g., Hodges, 2000).

Regarding possible limitations of the present study, it should be acknowledged that although we have demonstrated cases where providing schematic information did not enhance but rather impaired performance, we have not necessarily identified the precise mechanisms responsible. This should therefore be one focus for future work that would then allow us to predict *in advance* which forms or presentation methods of schematic knowledge hinder rather than help performance (as in Experiments 1 and 3) or appear to be redundant (as in Experiment 2). In addition, some of the present schematic information (e.g., in Experiment 1) may have been more useful if, for example, it had been available at retrieval as well as at encoding. Or perhaps the potentially interfering effect of schematic information (e.g., in Experiment 3) could have been minimized by presenting it in a different modality, such as verbally. Clearly, further investigations would be required to address these important remaining questions and possibilities.

In summary, prior research has shown that schematic information can reduce memory performance when to-be-remembered information is inconsistent with schematic knowledge (Alba & Hasher, 1983; Umanath & Marsh, 2014). Schema-consistent information can also be remembered less well than schema-inconsistent information when schema-inconsistent information stands out from a context, for example, in the isolation effect (Wallace, 1965) and other distinctiveness effects (Schmidt, 1991). The current data are novel in showing negative effects of schema activation without any schema-inconsistent conditions. The data show that schematic processing can come with a cost, especially for older adults, and that this cost can offset mnemonic benefits associated with schema-consistent processing of stimuli. Importantly, the current study reveals a factor that has been previously overlooked in studies of prior knowledge and aging (cf. Umanath & Marsh, 2014). Future studies investigating how knowledge/experience can influence age differences in memory therefore need to consider how accessible that knowledge is and whether it requires cognitive resources to use it effectively.

## Disclosure statement

No potential conflict of interest was reported by the authors.

## Notes

1. This excludes one young adult who did not follow instructions and one older adult whose data were gathered incorrectly due to a technical error.

2. The counterbalancing conditions of schema presence order (present–absent or vice versa) and encoding speed order (fast-slow or vice versa) showed no main effects (*F*s < 1). The two versions of the stimuli led to different performance levels, *F*(1, 60) = 4.67, *MSE* = 0.20, *p* < .05,  **= .07. None of the counterbalancing conditions interacted with age or schema presence so the following analyses were conducted without counterbalancing as a factor.

3. There was no main effect (*F* = 1.18) or any interactions (*F*s < 1.95) involving the counterbalanced factor of label condition order so the data were analyzed without this as a factor.

4. The study was not underpowered: Power analysis using G*power software (Faul, Erdfelder, Lang, & Buchner, 2007) was used to determine how many participants would be required to achieve a power of .8 to detect a medium effect size (as defined by Murphy & Myors, 1998) in the age by pair relatedness by label condition interaction. The study would have required 28 young and 28 older adults and this is fewer than the 31 young and 30 older participants tested.

5. These numbers exclude one young adult who did not follow instructions and one older adult whose data were lost during the experiment due to a computer crash.

6. There was no main effect of the order of the schema-present and schema-absent blocks (*F* < 1). This factor did interact with schema presence such that participants performed better with whichever condition they had first, *F*(1, 60) = 16.63, *MSE* = 0.02, *p* < .001,  **= .22, presumably due to interference in the latter test from the former and/or fatigue. However, the counterbalancing factor was not included in the main analysis, as it was not relevant to the hypotheses being tested.

## Appendix

**Table:** Word pairs used in Experiment 1 and their corresponding category relations (italic pairs were presented as buffers).

Left word	Right word	Relation (Schema label)	Left word	Right word	Relation (Schema label)
trespass	golfing	ACTIONS	athlete	sibling	PEOPLE
footwork	checkup	ACTIONS	locksmith	platoon	PEOPLE
crackdown	outburst	ACTIONS	classmate	cyclist	PEOPLE
courtship	longshot	ACTIONS	recluse	burglar	PEOPLE
handshake	issuance	ACTIONS	landlord	grandson	PEOPLE
eyesight	fracture	BODYPARTS/MEDICAL	*priestess*	*sidekick*	PEOPLE
frostbite	haircut	BODYPARTS/MEDICAL	causeway	vineyard	PHYSICAL LOCATIONS
*grimace*	*nostril*	BODYPARTS/MEDICAL	rooftop	parkland	PHYSICAL LOCATIONS
tendons	hygiene	BODYPARTS/MEDICAL	foothill	coastline	PHYSICAL LOCATIONS
ointment	thumbnail	BODYPARTS/MEDICAL	seashore	roadway	PHYSICAL LOCATIONS
medicines	whiplash	BODYPARTS/MEDICAL	*campsite*	*incline*	PHYSICAL LOCATIONS
farmhouse	sawmill	BUILDINGS	bramble	reptile	PLANTS/ANIMALS
racetrack	windmill	BUILDINGS	reindeer	grapevine	PLANTS/ANIMALS
showroom	courtyard	BUILDINGS	*rosebud*	*sparrow*	PLANTS/ANIMALS
cottage	nightclub	BUILDINGS	haystack	giraffe	PLANTS/ANIMALS
foundry	newsstand	BUILDINGS	cheetah	sunflower	PLANTS/ANIMALS
airfield	canteen	BUILDINGS	shamrock	hamster	PLANTS/ANIMALS
satchel	bracelet	FABRIC/WEARABLE	anguish	coolness	THOUGHTS/SENSATIONS
raincoat	bathrobe	FABRIC/WEARABLE	cowardice	numbness	THOUGHTS/SENSATIONS
footwear	knapsack	FABRIC/WEARABLE	*fondness*	*disgrace*	THOUGHTS/SENSATIONS
perfume	napkins	FABRIC/WEARABLE	firmness	willpower	THOUGHTS/SENSATIONS
swimsuit	washcloth	FABRIC/WEARABLE	dryness	foresight	THOUGHTS/SENSATIONS
*nightgown*	*textile*	FABRIC/WEARABLE	remorse	softness	THOUGHTS/SENSATIONS
appliance	glassware	HOUSEHOLD ITEMS	iceberg	teardrop	WATER
bathtub	staircase	HOUSEHOLD ITEMS	shipwreck	rainfall	WATER
hairbrush	doorbell	HOUSEHOLD ITEMS	snowstorm	spittle	WATER
armchair	briefcase	HOUSEHOLD ITEMS	snowflake	whirlpool	WATER
dustbin	lightbulb	HOUSEHOLD ITEMS	monsoon	driftwood	WATER
*aspirin*	*suitcase*	HOUSEHOLD ITEMS	yardstick	leaflet	WOOD/PAPER
handcuffs	corkscrew	METAL	headboard	scrapbook	WOOD/PAPER
trombone	cauldron	METAL	plywood	toothpick	WOOD/PAPER
*keyhole*	*trashcan*	METAL	hardwood	tabloid	WOOD/PAPER
horseshoe	doorknob	METAL	sawdust	charcoal	WOOD/PAPER
barbell	saucepan	METAL	parchment	trapdoor	WOOD/PAPER
